# Development and validation of the suicide risk score: a novel suicide risk prediction tool for patients with end-stage kidney disease

**DOI:** 10.1093/ckj/sfaf370

**Published:** 2025-12-08

**Authors:** Deok Gie Kim, Sung Hwa Kim, Dae Ryong Kang, Seoung Wan Nam, Jun Young Lee, Jinhee Lee

**Affiliations:** Department of Surgery, Yonsei University, Seoul, Korea; Department of Statistics, Yonsei University, Wonju College of Medicine Wonju, Korea; Department of Precision Medicine, Yonsei University, Wonju College of Medicine Wonju, Korea; Department of Rheumatology, Yonsei University, Wonju College of Medicine Wonju, Korea; Department of Nephrology, Comprehensive Kidney Disease Research Institute, Yonsei University, Wonju College of Medicine Wonju, Korea; Yonsei University Wonju College of Medicine, Center of Evidence Based Medicine, Wonju, Korea; Institute of Convergence Science, Yonsei University, Seoul, Korea; Department of Psychiatry, Yonsei University, Wonju College of Medicine Wonju, Korea

**Keywords:** depression, ESKD, suicide

## Abstract

**Background:**

Despite the high suicide rates among patients with end-stage kidney disease (ESKD), there is no suicide prediction model specifically designed for this vulnerable population. Herein, we aimed to develop and validate a novel suicide risk score for ESKD patients.

**Methods:**

We analyzed data from the National Health Insurance Service (NHIS) of South Korea, including 251 819 patients aged above 18 years diagnosed with ESKD between 2007 and 2022 in South Korea. The mean follow-up duration was 6.6 years. The cohort was randomly divided into derivation (70%) and validation (30%) sets. Using multivariate Cox proportional hazard regression, key variables were incorporated to develop the suicide risk score, which was converted into a 48-point scoring system, which is composed of easily identifiable clinical parameters.

**Results:**

Among 176 273 patients in the derivation cohort, 1126 (0.64%) patients committed suicide. The suicide risk score demonstrated moderate discrimination in both the derivation (C-statistic, 0.694) and validation (C-statistic, 0.709) cohorts, with good calibration. In the validation cohort, patients scoring below 16, 17–32 and 33–48 had predicted 10-year suicide risk of 0.2%, 1.2% and 7.7%, respectively, while the observed 10-year risk were 0.3%, 0.8% and 3.9%. These findings highlight the model’s ability to effectively stratify risk using routinely available clinical data.

**Conclusions:**

The suicide risk score is a significant advancement in suicide risk prediction for ESKD patients. It is based on simple, routinely collected clinical indicators and provides an actionable tool for risk stratification and early intervention in daily practice.

KEY LEARNING POINTS **What was known:**

- People with end-stage kidney disease (ESKD) face a higher risk of suicide than the general population, but until now there has not been a tool made specifically to spot which ESKD patients are most at risk.

**This study adds:**

- We used health records from South Korea’s National Health Insurance Service to look at 251 819 adults (age >18 years) who were diagnosed with ESKD between 2007 and 2022.
- By examining a range of simple medical and demographic factors (age, body mass index, hemoglobulin, gender, residental area, depression, insomnia, smoking, suicide attempt, anxiety disorder, psychosis, alcohol abuse and physical acitivity), we built a 48-point scoring system.

**Potential impact:**

- Suicide risk in patients with ESKD can be predicted using routinely collected clinical data.

## INTRODUCTION

Renal replacement therapy is essential for patients with end-stage kidney disease (ESKD), and regardless of the type of renal replacement therapy, these patients face physical limitations that reduce their quality of life [1]. Additionally, most patients with ESKD have multiple comorbidities [2]. These limitations necessitate family and social support, leading to an increase in psychiatric comorbidities and heightened stress levels, which are well-known risk factors for suicide [3–5]. Recently, an analysis of data from the United States Renal Data System (USRDS) reported that the suicide rate among patients with ESKD was 24.2 suicides per 100 000 patient-years, which is 84% higher than the rate in the general US population [4].

Suicide is a global health problem and is the ninth leading cause of death among individuals aged 10–64 years worldwide. According to a World Health Organization report, South Korea had the highest suicide rate in the world in 2019, with an incidence rate of 28.6 per 100 000 people [6]. Since suicide is a preventable condition, many suicide prediction models have been devised to help prevent it [7]. Through the application of machine learning, the prediction rates of these models have significantly improved. However, these models often rely heavily on survey-based indicators [7, 8]. Additionally, the complexity of these models poses limitations for practical use in the clinical setting, as they can be time-consuming and cumbersome to implement. Furthermore, to the best of our knowledge, no suicide prediction model specifically for patients with ESKD is currently available.

Therefore, the aim of this study was to develop a scoring model that can assess the suicide risk of patients with ESKD using data extracted from South Korea’s nationwide cohort study. By utilizing this model, we intend to easily measure the suicide risk in the clinical setting, allowing for early intervention for patients with a high suicide risk.

## MATERIALS AND METHODS

### Study population and source of data

**Figure 1:**
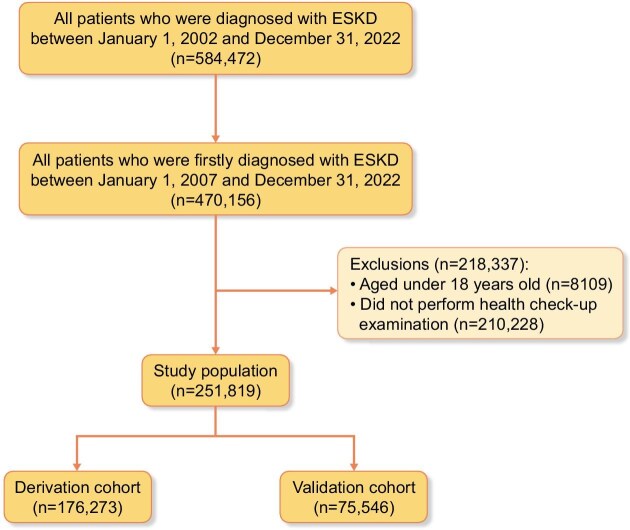
Flowchart depicting the selection process for patients with ESKD.

This study used data sourced from the National Health Insurance Service (NHIS) managed by the Ministry of Health and Welfare, Republic of Korea. In Korea, all hospitals must submit data on inpatient and outpatient visits, procedures, prescriptions and national health examinations to the NHIS. The NHIS assigns diagnosis codes using the International Classification of Disease, 10th edition. These data have been extensively validated in various studies [9]. The NHIS makes claims data available for research purposes, including mortality records detailing the cause and date of death, which are extracted from the Statistics Korea database (http://mdis.kostat.go.kr). Information on sensitive topics, such as suicide, is accessible through a distinct permission process from the Statistics Office. Researchers can access these data with the approval and supervision of the NHIS (NHIS-2019-1-343) via the Korean National Health Insurance Sharing Service (http://nhiss.nhis.or.kr). The specific codes used for diagnoses, procedures, and prescriptions examined in this study are listed in Supplementary data, Tables S1 and S2. Patients diagnosed with ESKD (*N* = 584 472) between 2002 and 2022 were selected. To determine the initial diagnosis of ESKD and utilize previous records, we established a washout period of 5 years and a minimal follow-up period of 1 year. Consequently, we identified 470 156 patients who were first diagnosed with ESKD between 2007 and 2022. We excluded 218 337 patients aged <18 years (*n* = 8109) or with no available health checkup records (*n* = 210 228). Finally, the cohort was randomly split into the derivation (70%, *n* = 176 273) and validation (30%, *n* = 75 546) cohorts (Fig. 1). The prediction index date was defined as the date of ESKD diagnosis, and all predictor variables were assessed using information available at or before this date. No-post ESKD information was used in model development.

### Study variables

To develop a model that can accurately predict suicide risk and prognosis in a clinical setting, we considered variables that are commonly used and easily recognized for patients with ESKD: age, sex, hemoglobin level, body mass index (BMI), physical activity, smoking history, alcohol abuse and history of psychiatric disease (suicide attempt, depression, insomnia, anxiety disorder and psychosis). The energy expenditure of physical activity was calculated as metabolic equivalent of tasks (METs), which is determined by adding frequency and intensity of physical activity [10–12].

### Development of the scoring system

To simplify the algorithm for practical application in a real clinical environment, continuous variables were categorized based on commonly used criteria. To select the appropriate variables for the scoring system, multivariable Cox proportional hazard regression using backward elimination was performed using significant variables obtained in the univariate analysis and multi-collinearity was confirmed [13]. Subsequently, the score of each predictor included in the scoring system was calculated using the β-coefficient in multivariate logistic regression analysis. Specifically, 1 point was assigned to the predictor with the smallest β-coefficient value. Scores for each predictor were rounded up by dividing the β-coefficient of each predictor by the smallest β-coefficient value. Points for each score category were assigned based on the regression coefficients of the multivariate model; the suicide risk score (range 0–48) was developed from these variables. To facilitate clinical interpretation, the suicide risk score was categorized into risk groups according to the suicide probability determined by Kaplan–Meier estimates.

### Validation and other statistical analysis

The model was evaluated using a competing risk analysis with mortality as a competing risk. The performance of the risk model was assessed using Harrell’s C-statistic penalized for optimism from the 200 bootstrapped resamples [14]. Model calibration was assessed using the Nam and D’Agostino goodness-of-fit test, and calibration plots were visually evaluated using 200 iterations [15]. All patients in the derivation cohort were considered by using the bootstrapping method, which is more robust than a validation method using split in internal validation [16]. We used analytic approaches and reporting standards as recommend by TRIPOD for risk prediction (Supplementary data, Table S3) [17]. All statistical analyses were performed using SAS statistical software version 9.4 (SAS Institute, Cary, NC, USA) and R version 4.3.0 (R Foundation for Statistical Computing, Vienna, Austria). A two-sided *P*-value <.05 indicated statistical significance.

## RESULTS

Baseline characteristics of the derivation and validation cohorts are presented in Supplementary data, Table S4. No significant differences were observed between the two cohorts.

### Model development population

Of 176 273 individuals included in the derivation cohort, 1126 (0.64%) committed suicide during the follow-up period. Individuals who committed suicide had a lower BMI (24.4 versus 23.6 kg/m^2^, *P <* .001), lower Charlson Comorbidity Index (CCI) score (5.1 versus 3.9, *P <* .001), and had a frequent psychiatric history [including depression, insomnia, suicide attempt, anxiety disorder, psychosis, substance misuse, post-traumatic stress disease (PTSD), bipolar disorder, schizophrenia]. These individuals also more frequently engaged in smoking and drinking. However, these patients had fewer medical comorbidities such as diabetes, hypertension, myocardial infarction and rheumatologic diseases. The detailed comparison of individuals with and without suicide events are presented in Table 1.

**Table 1:** Baseline characteristics of patients with suicide and non-suicide events (derivation cohort).

Variables	Non-suicide (*n* = 175 147)	Suicide (*n* = 1126)	*P*-value
Age, years	65.7 ± 13.5	65.2 ± 13.5	.3
BMI, kg/m^2^	24.4 ± 3.7	23.6 ± 3.2	<.001
Hemoglobin, g/dL	12.5 ± 2.1	13.1 ± 2	<.001
METs, MET-min/week	898.0 ± 1231.1	843.8 ± 1276.9	.4
Waist circumference, cm	84.7 ± 9.9	83.7 ± 9.2	.001
CCI score	5.1 ± 3.3	3.9 ± 3.2	<.001
Myocardial infarction	10 391 (5.9)	52 (4.6)	.06
Congestive heart failure	45 180 (25.8)	198 (17.6)	<.001
Peripheral vascular disease	46 241 (26.4)	265 (23.5)	.03
Cerebrovascular disease	40 128 (22.9)	239 (21.2)	.18
Dementia	18 724 (10.7)	80 (7.1)	<.001
Chronic pulmonary disease	65 716 (37.5)	396 (35.2)	.1
Rheumatologic disease	13 409 (7.7)	69 (6.1)	.05
Peptic ulcer disease	57 537 (32.9)	378 (33.6)	.6
Mild liver disease	73 219 (41.8)	441 (39.2)	.07
Diabetes without chronic complication	92 761 (53)	469 (41.7)	<.001
Diabetes with chronic complication	62 938 (35.9)	302 (26.8)	<.001
Hemiplegia, paraplegia	4870 (2.8)	23 (2)	.1
Malignancy without metastasis	32 492 (18.6)	147 (13.1)	<.001
Moderate, severe liver disease	4007 (2.3)	16 (1.4)	.05
Metastatic solid tumor	5460 (3.1)	15 (1.3)	.001
AIDS	249 (0.1)	0 (0.0)	.2
Age category			.8
<65 years	80 509 (46)	522 (46.4)	
≥65 years	94 638 (54)	604 (53.6)	
BMI category			<.01
<18.5 kg/m^2^	6417 (3.7)	47 (4.2)	
18.5–24.9 kg/m^2^	98 345 (56.2)	718 (63.8)	
25–29.9 kg/m^2^	58 569 (33.4)	332 (29.5)	
>30 kg/m^2^	11 816 (6.8)	29 (2.6)	
CCI category			<.001
≤7	136 843 (78.1)	968 (86)	
>7	38 304 (21.9)	158 (14)	
Hemoglobin category			<0.001
≤10 g/dL	22 916 (13.1)	56 (6.7)	
>10 g/dL	152 231 (86.9)	1039 (92.3)	
Sex			<.001
Male	104 057 (59.4)	828 (73.5)	
Female	71 090 (40.6)	298 (26.5)	
Household income			.7
Quantile 1	42 476 (24.3)	264 (23.5)	
Quantile 2	30 282 (17.3)	194 (17.2)	
Quantile 3	39 425 (22.5)	269 (23.9)	
Quantile 4	62 964 (36)	399 (35.4)	
Residential area			<.001
Urban	100 440 (57.4)	787 (69.9)	
Rural	74 707 (42.7)	339 (30.1)	
History of hospitalization	124 511 (71.1)	790 (70.2)	.5
History of ED visit	41 620 (23.8)	234 (20.8)	.02
Psychiatric clinic	3 248 (1.9)	27 (2.4)	.2
Depression	74 901 (42.8)	602 (53.5)	<.001
Insomnia	64 522 (36.8)	539 (47.9)	<.001
Suicide attempt	2361 (1.4)	27 (2.4)	.002
Atrial fibrillation	12 341 (7.1)	48 (4.3)	<.001
Smoking	32 534 (18.6)	294 (26.1)	<.001
Drinking	48 764 (27.8)	397 (35.3)	<.001
Diabetes mellitus	139 064 (79.4)	755 (67.1)	<.001
Hypertension	115 789 (89.3)	602 (71.8)	<.001
Anxiety disorder	89 444 (51.1)	653 (58)	<.001
Psychosis	7079 (4)	82 (7.3)	<.001
Substance misuse	7433 (4.2)	122 (10.8)	<.001
PTSD	389 (0.2)	6 (0.5)	.03
Bipolar disorder	11 141 (6.4)	96 (8.5)	.003
Schizophrenia, schizophrenic affective disorder	4422 (2.5)	53 (4.7)	<.001
Amputation	2421 (1.4)	11 (1)	.2
Alcohol abuse	682 (0.4)	21 (1.9)	<.001
Drug abuse	28 944 (16.5)	274 (24.3)	<.001
CRPS	3570 (2)	19 (1.7)	.4
Myocardial infarction	24 116 (13.8)	124 (11)	.007
Stroke	49 856 (28.5)	300 (26.6)	.2
MACE	62 830 (35.9)	374 (33.2)	.06
Liver cirrhosis	17 078 (9.8)	95 (8.4)	.1

Data are presented as mean ± standard deviation or *n* (%).

AIDS, acquired immune deficiency syndrome; CRPS, complex regional pain syndrome; ED, emergency department; MACE, major adverse cardiovascular event.

### Suicide risk prediction model

Univariate hazard ratio and C-index values are presented in Supplementary data, Table S5. In the univariate analysis, age, physical activity, high BMI, hemoglobin level, sex, residential area, history of hospitalization, history of emergency department (ED) admission, depression, insomnia, suicide attempt, smoking, drinking, diabetes, hypertension, anxiety disorder, psychosis, substance misuse, PTSD, bipolar disorder, schizophrenia, alcohol abuse and drug abuse were associated with suicide. The final model had a moderate discrimination in the derivation cohort over the study period {C-statistic, 0.694 [95% confidence interval (CI) 0.685–0.703]}. After internal validation with bootstrapping, the discrimination remained similar (C-statistic, 0.709 [95% CI 0.697–0.721]). The discrimination of the suicide risk score in patients undergoing hemodialysis [C-statistic, 0.708 (95% CI 0.69–0.720)] was superior to that of patients undergoing peritoneal dialysis [C-statistic, 0.627 (95% CI 0.498–0.756)] (Table 2) and similar in various time periods (Supplementary data, Table S6).

**Table 2:** Predictive performance of the suicide risk score for patients with ESKD.

	Optimism corrected C-index	95% CI
Derivation cohort	0.694	0.685–0.703
Validation cohort	0.709	0.697–0.721
HD	0.708	0.696–0.720
PD	0.627	0.498–0.756
KT	0.704	0.618–0.790

PD, peritoneal dialysis; KT, kidney transplantation.

The model was well-calibrated based on the visual inspection of calibration plots (shown in Supplementary data, Fig. S1; goodness-of-fit *P* = .71).

### Clinical risk prediction tool: the suicide risk score

Points were assigned to each variable in the model proportional to the model coefficients, with a maximum allocation of 48 points per risk factor. The points were assigned to age (≥65 years, 1 point), BMI (<18.5 kg/m^2^, 8 points; 18.5–24.9 kg/m^2^, 7 points; 25–29.9 kg/m^2^, 5 points; >30 kg/m^2^, 0 points), hemoglobin level (>10 g/dL, 4 points), sex (male, 5 points), residential area (rural, 4 points), depression (3 points), insomnia (3 points), smoking (2 points), suicide attempt (4 points), anxiety disorder (1 point), psychosis (3 points), alcohol abuse (9 points) and physical activity (METs >0, 1 point) (Table 3). By simplifying parameters to those easily detectable in clinical settings, two additional suicide risk scores were also developed, which demonstrated similar AUC values (Supplementary data, Tables S7 and 8).

**Table 3:** Suicide risk score.

Clinical risk prediction tool	Scores^a^
Age	
<65 years	0
≥65 years	1
BMI	
<18.5 kg/m^2^	8
18.5–24.9 kg/m^2^	7
25–29.9 kg/m^2^	5
>30 kg/m^2^	0
Hb category	
≤10 g/dL	0
>10 g/dL	4
Sex	
Male	5
Female	0
Residential area	
Urban	0
Rural	4
Depression	3
Insomnia	3
Smoking	2
Suicide attempt	4
Anxiety disorder	1
Psychosis	3
Alcohol abuse	9
Physical activity (no)	1

a Total score range: 0–48. Presented is the final clinical risk prediction scoring system, suicide risk score, assignment is based on patient characteristics. The prediction score was developed using the NHIS dataset and included patients who received renal replacement therapy in Korea. Points assignment was devised based on the coefficients of these variables in a Cox regression model for the outcome of completed suicide.

Hb, hemoglobin.

### Clinical risk groups

**Figure 2:**
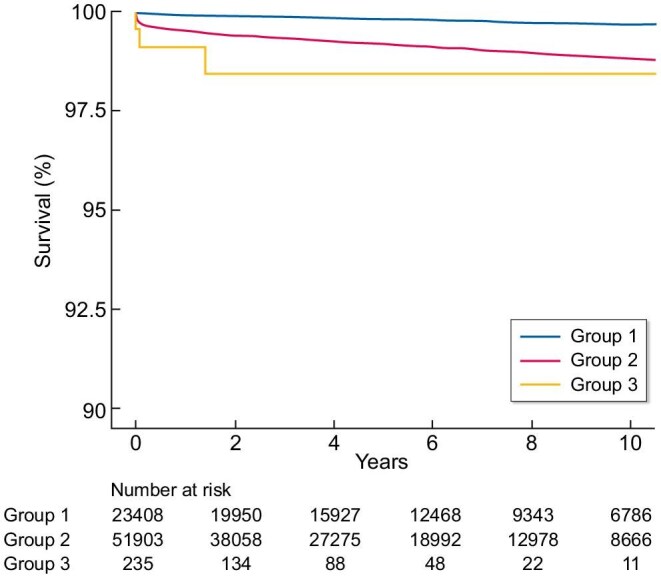
Kaplan–Meier curve for patient survival in the validation cohort. Presented are the cumulative suicide-free survival rates for individuals in Korean patients with ESKD. Cumulative survival curves were based on the risk category assigned to individuals by the suicide risk score. The risk score ranges from 0 to 48, with risk categories assigned as Group 1 (scores 0–16), Group 2 (scores 17–32) and Group 3 (score 33–48).

**Figure 3:**
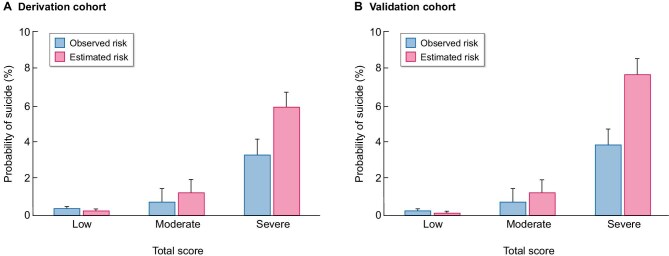
Comparison of risk of suicide according to the scoring model for observed risk and estimated risk.

Risk scores determined by the suicide risk score were categorized into clinical risk groups on the basis of observed 10-year suicide rates: low risk (<16 points, <0.3% estimated probability of suicide), moderate risk (17–32 points, 0.3%–1.2% estimated probability of suicide) and high risk (>33 points, >5.9% estimated probability of suicide). For each higher clinical risk group, there was an incremental increase in the risk of suicide in both the derivation and validation cohorts (Fig. 2). For the derivation and validation cohorts, the actual prevalence of suicide events and predicted prevalence of suicide according to the suicide risk score are shown Fig. 3. A strong correlation was observed between the observed and estimated risks (r = 0.997, R^2^ = 0.994, *P* < .001) (Supplementary data, Fig. S1).

## DISCUSSION

In this study, we developed a novel scoring system to predict suicide risk among patients with ESKD. Our findings indicate that the suicide risk score had a moderate predictive ability, with a C-statistic of 0.694 in the derivation cohort and 0.709 after internal validation. This model stands out due to its focus on patients with ESKD, filling a critical gap in existing suicide prediction models. Importantly, this is a prognostic model intended for risk prediction rather than for causal inference about the included predictors.

The Beak Scale for Suicide Ideation [18] and Columbia–Suicide Severity Rating Scale (C-SSRS) [19] are two representative suicide risk assessment tools. However, both scales require assessment by mental health professionals, especially psychiatrists and psychologists, and take a considerable amount of time to evaluate risk. Recently, many suicide prediction models for suicide death have been developed using electronic medical records [20–25]. However, these prediction models have limited clinical value, not because of their low sensitivity and positive predictive value, but due to the lack of information on the effectiveness of the targeted clinical interventions. Efforts to develop and evaluate suicide prediction tools should focus on specific clinical decisions and assess their value based on net benefit to the patient [7, 8]. The suicide risk score developed in the present study differs from these previous models owing to its exclusive application for patients with ESKD and integration of 16 diverse predictors, including clinical, demographic and psychiatric variables. Unlike other models that often rely heavily on survey-based indicators, which require well trained psychiatric professionals and require more time to complete, the suicide risk score uses readily available clinical data and employs a scoring system, making it more practical for routine use in the clinical setting.

In general, a history of psychiatric disorders has been associated with an increased risk of suicide [21, 22]. In patients with ESKD, assessing psychiatric history, alcohol consumption and smoking status is crucial for predicting suicide risk [26]. This approach is considered similar to that used in the general population. Therefore, psychiatric history should be carefully considered in patients with ESKD in clinical settings to predict the risk of suicide effectively.

However, unlike patterns typically reported in the general population, medical conditions such as diabetes in patients with ESKD were associated with a lower risk of suicide in our model and in an analysis of USRDS data (hazard ratio 0.76, 95% CI 0.59–0.99) [4, 27].

This finding should be interpreted as a prognostic association rather than as evidence of a causal protective effect, and the mechanisms underlying this pattern remain uncertain. Although the exact reasons for this association are unclear, several non-causal explanations have been proposed for this inverse relationship. First, patients with ESKD who have multiple comorbidities (such as hypertension, diabetes or a higher CCI score) often have a higher risk of cardiovascular events and all-cause mortality, leading to reduced long-term survival. This may introduce survivorship bias—in which patients with severe comorbidities are less likely to survive long enough to be included in analyses of suicide risk, thereby suggesting that medical comorbidities are associated with a lower suicide risk. Second, these patients require more frequent hospital visits and closer interactions with healthcare providers. This increased medical attention may facilitate more proactive disease management and provide greater opportunities for social and emotional support, potentially creating a more protective care context without implying that the comorbidities themselves are protective. Lastly, patients with multiple comorbidities may experience significant physical functional decline and psychological energy depletion, which could limit their ability or motivation to engage in suicidal behaviors. For patients with ESKD, the cumulative strain of managing several chronic illnesses alongside the dialysis regimen may result in reduced autonomy and vitality, which could further contribute to the observed lower incidence of suicide in this population. These explanations are speculative and highlight that the observed association is likely to reflect underlying complexity rather than a simple causal effect.

In clinical practice, the suicide risk score may help nephrologists and dialysis clinicians move beyond a general awareness that psychiatric comorbidities are associated with increased risk, by providing a simple, ESKD-specific tool that synthesizes multiple routinely collected factors into an absolute risk estimate. Patients identified in the highest risk category could be prioritized for structured suicide risk assessment, early psychiatric referral and enhanced psychosocial support, whereas those in lower risk groups may be managed with standard monitoring. Because all predictors are derived from routine health examinations and claims data, the score can be implemented without additional questionnaires or specialist evaluation and may be integrated into electronic health record systems to enable automated risk stratification at the point of care, pending external validation.

This study has some limitations. First, the risk score was primarily validated for individuals who had committed suicide; however, the decision to treat patients with psychiatric disorders often requires weighing the impact and severity of different types of psychiatric diseases. Second, validation using suicide rate was limited by the low number of events, although we observed that the suicide risk score had similar predictive ability to the observed and estimated suicide risks. Third, information on other potential covariates such as educational level and occupation was missing because this was not available in the NHIS dataset. Fourth, we could not investigate suicidal ideation and psychiatric disease–related questionnaires. We included death confirmation codes with intentional self-harm, which indicated successful suicide attempts; however, we could not include failed suicide attempts. Fifth, because the risk score was developed from observational claims data, the identified predictors should be interpreted as prognostic markers rather than causal determinants of suicide risk. Sixth, our model was developed and validated in an incident ESKD cohort, with all predictors assessed at the time of diagnosis. Therefore, its predictive accuracy in prevalent patients who have been on renal replacement therapy for many years (e.g. >5 or 10 years) is uncertain, as their clinical characteristics and risk profiles may differ substantially from those at the time of diagnosis. Future studies evaluating the performance and calibration of this risk score in prevalent ESKD populations are warranted. Additionally, because patients without health screening data could not be evaluated further due to structural constraints within the NHIS database, their exclusion may introduce some degree of selection bias. Given the long study period (2002–22), we performed a sensitivity analysis separating early and late periods; the discrimination of the score remained stable between 2002–12 and 2013–22 (C-indices 0.712 and 0.695, respectively), indicating that temporal changes in practice patterns exerted minimal influence on model performance. Furthermore, suicide deaths were identified using official cause-of-death codes assigned by Statistics Korea, which have shown high validity in prior evaluations, suggesting that misclassification of suicide outcomes is likely to be limited.

Nevertheless, to best of our knowledge, this is the first suicide risk scoring model for patients with ESKD, which enables clinicians to rapidly predict suicide risk within a few minutes simply through history taking.

In conclusion, we developed a suicide risk score for assessing suicide risk in patients with ESKD. Without the need for additional psychiatric disease assessment tools, the proposed suicide risk score predicts suicide risk with easily accessible variables.

## Supplementary Material

sfaf370_Supplemental_File

## Data Availability

The data that support the findings of this study are available from the corresponding author upon reasonable request.
