# Supplementary material for: Development and validation of the suicide risk score: a novel suicide risk prediction tool for patients with end-stage kidney disease
Source: Clin Kidney J. 2025 Dec 8;19(2):sfaf370. doi: 10.1093/ckj/sfaf370 (PMC12863073; doi:10.1093/ckj/sfaf370)
Supplement: sfaf370_Supplemental_File [file sfaf370_supplemental_file.docx]

**Supplementary data**

**Table of Contents**

**Table S1:** Diagnoses, procedures, and specific codes.

**Table S2:** Drugs (code and dose) reported in the National Health Insurance Korea database analysis.

**Table S3:** TRIPOD Checklist.

**Table S4:** Baseline characteristics of the derivation and validation cohorts.

**Table S5:** Univariate hazard ratio and C-index for suicide risk by variables.

**Table S6.** Predictive performance of the suicide risk score for patients with ESKD.

**Table S7:** The other suicide risk scores using other variables (AUC: 0.694).

**Table S8:** The other suicide risk score using minimal variables (AUC: 0.681).

**Figure S1:** Calibration plot for suicide.

**Table S1.** Diagnoses, procedures, and specific codes.

| Disorder | Diagnosis, procedure, specific codes^a^ |
| --- | --- |
| Acquired immunodeficiency syndrome | B20 B21, B22, B24 |
| Alcohol abuse | F10, E52, G62.1, I42.6, K29.2, K70.0, K70.3, K70.9, T51.x, Z50.2, Z71.4, Z72.1 |
| Anxiety disorder | F40, F41 |
| Amputation | N0573, N0574, N0571-5, N0562, N0564, N0565, N0566, Y835 |
| Atrial fibrillation | I480, I482, I4891 |
| Bipolar disorder | F31 |
| Cancer | C00 to C97 |
| Cerebrovascular disease | G45, G46, I60, I61, I62, I63, I64, I65, I66, I67, I68, I69, H340 |
| Chronic lung disease | J40, J41, J42, J43, J44, J45, J46, J47, J60, J61, J62, J63, J64, J65, J66, J67, I278, I279, J684, J701, J703 |
| CRPS | G90.5 |
| Diabetes | E11 to E14 or ≥ 1 antidiabetic medication |
| Dementia | F00, F01, F02, F03, G30, G311, F051 |
| Depression | F32.x, F33.x, F34.1 |
| Drug Abuse | F11.x - F16.x, F18.x, F19.x, Z71.5, Z72.2 |
| Heart failure | I43, I50, I099, I110, I130, I132, I255, I420, I425, I426, I427, I428, I429, I97.1, P290 |
| Hemiplegia | G81, G82, G041, G114, G801, G802, G830, G831, G832, G833, G834, G839 |
| Hemodialysis | Z491, N185, I120, Z992, O7020, O7021, V001^a^ |
| Hypertension | I10, I11, I12, I13, I15 or ≥ 1 antihypertensive medication |
| ICU Admission | AJ001, AJ003, AJ004, AJ005, AJ006, AJ007, AJ008, AJ009, AJ010, AJ011, AJ020, AJ021, AJ031, AJ041, AJ042, AJ043, AJ044, AJ045, AJ046, AJ100, AJ101, AJ102, AJ103, AJ110, AJ111, AJ112, AJ120, AJ121, AJ122, AJ130, AJ131, AJ132, AJ140, AJ141, AJ142, AJ143, AJ144, AJ150, AJ151, AJ152, AJ160, AJ161, AJ180, AJ190, AJ200, AJ201, AJ202, AJ203, AJ210, AJ211, AJ212, AJ220, AJ221, AJ222, AJ230, AJ231, AJ232, AJ240, AJ241, AJ242, AJ244, AJ250, AJ251, AJ252, AJ260, AJ261, AJ280, AJ290, AJ300, AJ301, AJ302, AJ303, AJ310, AJ311, AJ312, AJ320, AJ321, AJ322, AJ330, AJ331, AJ332, AJ340, AJ341, AJ342, AJ350, AJ351, AJ352, AJ360, AJ380, AJ390, AJ500, AJ510, AJ520, AJ530, AJ540, AJ550, AJ560, AJ580, AJ590 |
| Insomnia | G47, F510 |
| Liver cirrhosis | K74.60 |
| MACE | Composite of cardiovascular mortality, acute myocardial infarction, and stroke |
| Malignancy without metastasis | C00 to C97 (except C77, C78, C79, C80) |
| Metastatic solid tumor | C77, C78, C79, C80 |
| Mild liver disease | B18, K73, K74, K700, K701, K702, K703, K709, K713, K714, K715, K717, K760, K762 K763, K764, K768, K769, Z944 |
| Moderate-to-severe liver disease | I850, I859, I864, I982, K704, K711, K721, K729, K765, K766, K767, K704, K711 |
| Myocardial infarction | I21, I22, I252 |
| Peptic ulcer disease | K25, K26, K27, K28 |
| Peripheral vascular disease | I700 to I702, I708, I709, K551, K558, K559, Z958, Z959, I1731, I1738, I1739, I1771, I1790, I1792 |
| Peritoneal dialysis | Z492, O7061, O7062, O7071, O7072, O7074, O7076, O7077, V003^a^ |
| Psychosis | F20.x, F22.x - F25.x, F28.x, F29.x, F30.2, F31.2, F31.5 |
| PTSD | F431 |
| Rheumatologic disease | M05, M06, M32, M33, M34, M315, M351, M353, M360 |
| Vascular disease | I20, I21, I22, I24, I25 |
| Stroke | I60–63 and admitted more than two days |
| Schizophrenia, Schizophrenic affective disorder | F20, F25 |
| Substance misuse | F10–19 |
| Suicide attempt | X60–84, Y87, R458, z915 |

All diagnostic, procedural, and specific codes are available at <https://www.hira.or.kr/>.
PTSD, post-traumatic stress disorder; ICU, intensive care unit; CRPS, complex regional pain syndrome; MACE, major adverse cardiovascular event.

^a^Specific code: With this code, patients on dialysis can receive a certificate of disability and an additional insurance discount.

In this study, the requested and approved NHIS data were merged with the data from the mortality records database of Statistics Korea (http://mdis.kostat.go.kr), which includes the cause and date of mortality. ICD-10 codes were used in the analyses.

**Table S2.** Drugs (code and dose) reported in the National Health Insurance Korea database analysis.

| Drug | Health Insurance Review & Assessment (HIRA) service code |
| --- | --- |
| ACEi or ARBs or aliskiren | 104201ATB, 104202ATB, 114701ATB, 122601ATB, 122602ATB, 122603ATB, 122901ATB, 122902ATB, 122903ATB, 133001ATB, 133002ATB, 133003ATB,  140901ATB, 140902ATB, 151601ATB, 151603ATB, 163501ATB, 163502ATB, 104201ATB, 104202ATB,114701ATB, 122601ATB, 122602ATB, 122603ATB, 122901ATB, 122902ATB, 122903ATB, 133001ATB, 133002ATB, 133003ATB, 140901ATB, 140902ATB, 151601ATB, 151603ATB, 163501ATB, 163502ATB, 173401ATB, 173402ATB, 177301ATB, 177303ATB, 177303ATB, 184501ATB, 185701ATB, 185702ATB, 196801ATB, 196802ATB, 211301ATB, 211302ATB, 221901ATB, 222401ACH, 222401ATB, 222402ACH, 222402ATB, 222404ATB, 235002ATB, 247101ATB, 247102ATB, 247103ATB, 247104ATB, 378801ATB, 378802ATB, 378803ATB, 429201ATB, 468501ATB, 468502ATB, 468503ATB, 501601ATB, 501602ATB, 510401ATB, 510402ATB, 510403ATB, 515201ATB, 515202ATB, 515203ATB, 520901ATB, 520902ATB, 662401ATB, 662402ATB, 662403ATB, 651401ATB, 651402ATB, 651403ATB |
| BBs | 117903ATB, 117904ATB, 124801ATB, 219901ATB, 219902BIJ, 219904ATB, 219905ACR, 219906ACR, 111401ATB, 111402ATB, 111403ATB, 116801ATB, 116803ATB, 117001ATB, 117002ATB, 125001ATB, 125002ATB, 125003ATB, 125004ACR, 125005ATB, 125006ACR, 125007ACR, 125007ATR, 125008ACR, 125008ATR, 483101ATB, 483102ATB, 489501ATB, 489502ATB, 489503ATB, 662201ATB, 662202ATB, 117901ATB, 117902ATB, 129101ATB, 193802ATB, 194003ATR, 198301ATB, 154401BIJ, 154402BIJ, 154431BIJ, 154430BIJ, 180201ATB, 180201BIJ, 180202BIJ, 180230BIJ, 180231BIJ |
| CCBs | 107601ATB, 107601ATD, 107602ATB, 107602ATD, 107603ATB, 114001ACH, 114002ACH, 114003ACH, 115101ATB, 115102ATB, 115103ATB, 115104ATB, 133101ATB, 133102ATB, 157501ATR, 157503ATR, 178902ACR, 180301ATB, 180302ATB, 180303ATB, 182001ATB, 182002ATB, 188001ATB, 188002ATB, 201001BIJ, 201002ATB, 201002BIJ, 201003ACR, 201030BIJ, 201031BIJ, 201033BIJ, 202401ATB, 202402ACS, 202402ATB, 247601ACR, 247603ATR, 247604BIJ, 247605ATR, 247606ATB, 247607ATB, 459801ACH, 459801ATB, 459802ACH, 459901ATB, 459902ATB, 464601ATB, 470801ATB, 470802ATB, 476201ATB, 479701ATB, 483201ATB, 483202ATB, 489501ATB, 489502ATB, 489503ATB, 145702BIJ, 145703ACR, 145704BIJ, 145706ATB, 145706ATR, 145707ACR, 145707ATB, 145707ATR, 201401ACS, 201401ATB, 201402ATB, 201405ATR, 201407ACS, 201408ATR, 201409ATR, 201702ATB, 201901ATB, 201902BIJ, 201930BIJ, 356201ATB, 356202ATB, 356202ATR, 356203ATR, 441201ATB, 441202ATB, 528201ATR, 528202ATR, 145702BIJ, 247630BIJ, 486501ATB, 486502ATB, 495901ATB, 501701ATB |
| Diuretics | 101501ATB, 101502BIJ, 106901ATB, 163801ATB, 163802BIJ, 163830BIJ, 170801ATB, 174401ATR, 174402ATB, 174403ATB, 231101ATB, 231102ATB, 244701ATB, 262700ATB, 367001ATB, 367002ATB, 451301ATB, 451302ATB |
| ACEi or ARBs and CCBs | 447100ATB, 447200ATB, 466000ATB, 492800ATB, 492900ATB, 495800ATB, 500500ATB, 500600ATB, 582200ATB, 582400ATB, 502700ATB, 503000ATB, 513900ATB, 511500ATB, 511600ATB, 511700ATB, 623100ATB, 521200ATB, 521300ATB, 521400ATB, 644800ATB, 522200ATB, 522300ATB, 522400ATB, 522600ATB, 522700ATB, 522800ATB, 522900ATB, 523000ATB, 523100ATB, 523200ATB, 523300ATB, 523400ATB, 547500ATB, 547600ATB, 547700ATB, 547800ATB, 547900ATB, 548000ATB, 631300ATB, 629400ATB, 629500ATB, 629600ATB, 632800ATB, 632900ATB, 633000ATB, 637400ATB, 637500ATB, 637600ATB, 644800ATB, 651900ATB, 652000ATB, 652100ATB, 652700ATB, 652900ATB, 653000ATB, 653100ATB |
| ACEi or ARBs and statins | 524000ATB, 524100ATB, 527000ATB, 527100ATB, 525000ATB, 525100ATB, 525200ATB, 525300ATB, 629700ATB, 629800ATB, 526300ATB, 526400ATB, 526500ATB, 526900ATB, 644100ATB, 644200ATB, 653200ATB, 629900ATB, 630000ATB, 630100ATB, 630200ATB, 631600ATB, 631700ATB, 634900ATB, 635000ATB, 635100ATB, 635200ATB, 653200ATB, 654600ATB, 654700ATB, 654800ATB, 654900ATB, 655000ATB, 661800ATB, 661900ATB, 662000ATB, 662100ATB, 673700ATB, 688100ATB, 688200ATB, 688300ATB, 688400ATB, 688500ATB |
| BB with diuretics | 262100ATB, 262600ATB, 460200ATB, 469800ATB, 469900ATB, 470000ATB |
| ACEi or ARBs and diuretics | 262200ATB, 262300ATB, 262500ATB, 378900ATB, 440300ATB, 453600ATB, 453700ATB, 486900ATB, 356400ATB, 442600ATB, 385700ATB, 385800ATB, 423700ATB, 440800ATB, 443200ATB, 443300ATB, 502600ATB, 448600ATB, 448700ATB, 460500ATB, 477400ATB, 490100ATB, 497900ATB, 499200ATB, 499300ATB, 513600ATB, 522000ATB, 526800ATB, 556200ATB, 673500ATB, 673600ATB |
| ACEi or ARBs and CCBs and diuretics | 519700ATB, 519800ATB, 519900ATB, 520000ATB, 520100ATB, 662800ATB, 662900ATB, 663000ATB, 663500ATB, 663600ATB, 663700ATB, 663800ATB, 682700ATB, 682800ATB, 682900ATB |
| ACEi or ARBs and CCBs and statins | 663900ATB, 664000ATB, 664100ATB, 664200ATB, 664300ATB, 664400ATB, 671200ATB, 671300ATB, 671400ATB, 671500ATB, 671600ATB, 671700ATB, 677000ATB, 677100ATB, 677300ATB, 677400ATB, 677500ATB, 677600ATB, 686800ATB, 679500ATB, 679600ATB, 679700ATB, 680300ATB, 684300ATB, 684400ATB, 684500ATB, 684600ATB, 684700ATB, 686800ATB, 686900ATB, 690400ATB, 690500ATB, 690600ATB, 690700ATB, 691400ATB, 691500ATB |
| CCBs and statins | 472300ATB, 472400ATB, 472500ATB, 518900ATB, 614500ATB, 673900ATB, 674000ATB, 674100ATB, 678600ATB |
| BBs and CCBs | 262400ATR |
| BBs and statins | 683000ATB, 683100ATB, 683200ATB, 691200ATB |
| Alpha blockers | 149101ATB, 149102ATB, 149104ATR, 483401ACH, 104803ATR, 159001ATB, 234601ACR, 234601ATD, 234601ATR, 234602ACR, 234603ACR, 234603ATD, 234603ATR, 235501ATB, 235502ATB, 235503ATB, 458801ACS, 458801ATB, 504201ACH, 504202ACH, 504202ATB, 504203ACH, 504203ATD, 505801ATB, 505802ATD, 614201ATB, 614202ATB, 614203ATB |
| Statins or ezetimibe or fibrate | 111501ATB, 111502ATB, 111503ATB, 111504ATB, 162401ACH, 162402ACH, 162403ATR, 185801ATB, 216601ATB, 216602ATB, 216603ATB, 216604ATB, 218001ATB, 227801ATB, 227801ATR, 227802ATB, 227803ATB, 227805ATB, 227806ATB, 454001ATB, 454002ATB, 454003ATB, 462201ATB, 470901ATB, 470902ATB, 470903ATB, 471000ATB, 471100ATB, 507800ATB, 502201ATB, 502202ATB, 502203ATB, 502204ATB, 519300ACH, 631400ATB, 631500ATB, 633800ATB, 633900ATB, 634600ATB, 634800ATB, 640700ATB, 640800ATB, 640900ATB, 663400ACS, 679300ACH |
| DM medications | 170101BIJ, 170102BIJ, 170103BIJ, 170130BIJ, 170131BIJ, 170401BIJ, 170402BIJ, 170430BIJ, 170431BIJ, 170502BIJ, 175301BIJ, 175302BIJ, 175304BIJ, 175330BIJ, 175331BIJ, 175332BIJ, 175333BIJ, 441301BIJ, 441302BIJ, 441303BIJ, 441304BIJ, 441305BIJ, 441330BIJ, 441331BIJ, 441332BIJ, 441333BIJ, 441334BIJ, 461801BIJ, 461802BIJ, 461804BIJ, 461830BIJ, 461831BIJ, 461832BIJ, 484901BIJ, 484902BIJ, 484930BIJ, 484931BIJ, 488701BIJ, 488730BIJ, 507401BIJ, 626700BIJ, 626801BIJ, 626802BIJ, 626830BIJ, 626831BIJ, 512101BIJ, 512102BIJ, 512130BIJ, 512131BIJ, 626601BIJ, 626602BIJ, 626630BIJ, 626631BIJ, 639701BIJ, 639702BIJ, 644501BIJ, 644502BIJ, 666700BIJ, 667000BIJ, 527301ATB, 527302ATB, 628201ATB, 628202ATB, 636101ATB, 639800ATR, 641400ATR, 649000ATB, 649100ATB, 649200ATB, 649300ATB, 649400ATB, 649500ATB, 674301ATB, 674302ATB, 100601ATB, 100602ATB, 165402ATB, 165601ACS, 165602ACS, 165602ATB, 165603ATR, 165604ATR, 165701ATB, 165702ATB, 165703ATB, 165704ATB, 165801ATB, 191501ATB, 191502AGR, 191502ATB, 191502ATR, 191503ATB, 191504ATB, 191504ATR, 191505ATR, 249001ATB, 249002ATB, 249002ATD, 348002ATB, 379501ATB, 379502ATB, 379503ATB, 406201ATB, 406202ATB, 421100ATB, 430201ATB, 430202ATB, 430203ATB, 431901ATB, 431902ATB, 443400ATB, 443500ATB, 452700ATB, 452900ATB, 469100ATB, 471900ATB, 474200ATB, 474300ATB, 474300ATR, 488800ATB, 488900ATB, 489000ATB, 498100ATB, 498600ATB, 486101ATB, 497200ATB, 498100ATB, 523600ATB, 523700ATB, 525500ATB, 525600ATB, 525901ATB, 631900ATB, 632100ATB, 637200ATB, 653800ATR, 653900ATR, 654000ATR, 655700ATR, 518800ATB, 500801ATB, 501101ATB, 501102ATB, 501103ATB, 502200ATB, 502300ATB, 502300ATR, 502900ATB, 513700ATB, 513700ATR, 524700ATR, 507000ATB, 507100ATB, 519600ATB, 518500ATR, 518600ATR, 520500ATB, 520600ATB, 520700ATB, 523800ATR, 632000ATR, 645000ATR, 654100ATR, 613301ATB, 613302ATB, 616401ATB, 619101ATB, 624201ATB, 624202ATB, 624203ATB, 627301ATB, 630300ATB, 630400ATB, 630500ATB, 630600ATB, 635600ATB, 635700ATB, 675500ATB, 639601ATB, 641800ATR, 641900ATR, 642000ATR, 645301ATB, 648400ATB, 648500ATB, 648600ATB, 649900ATR, 650000ATR, 650100ATR |
| DM medications and statins | 664600ATB, 664700ATB, 664800ATB, 671800ATR, 673800ATR, 671900ATR, 672000ATR, 672100ATR, 672500ATR, 672600ATR, 672700ATR, 672800ATR, 672900ATR, 673000ATR, 683300ATR, 683400ATR |
| MAOi | 196701ATB, 226401ATB |
| NaSSA | 196201ATD, 196201ATB, 196202ATB, 196202ATD, 196204ATB, 196204ATD |
| NRI | 428101ATB, 428102ATR, 428103ATR |
| SARI | 242901ACH, 242901ATB, 242902ATB, 242903ATR |
| SNRI | 247502ACR, 247502ATR, 247504ACR, 247504ATR, 355801ACH, 355802ACH, 355803ACH, 495501ACE, 4995501ACH, 495501ATB, 499501ATE, 495502ACE, 499502ACH, 499502ATB, 495502ATE, 626401ATR, 626402ATR, 687601ATR, 687602ATR, 687701ATR, 687702ATR |
| SSRIs | 36301ACH, 136302ACH, 161501ACH, 161501ATB, 161502ACH, 161502ATB, 161502ATD, 209301ATB, 209302ATB, 209304ATR, 209305ATR, 227001ATB, 227002ATB, 227003ATB, 495501ACE, 495501ATE, 495502ACE, 495502ATE, 107501ATB, 107502ATB, 107504ATB, 162501ATB, 162502ATB, 173701ATB, 247502ACR, 247504ACR, 428301ATB, 474801ATB, 474802ATB, 474803ATB, 474804ATB, 149203ATB, 149204ATB, 108002ATB, 196201ATD, 196202ATB, 196202ATD, 196204ATB, 196204ATD, 196701ATB, 613101ATB |
| TCA | 149203ATB, 149204ATB, 107501ATB, 107502ATB, 107504ATB, 108002ATB, 136301ACH, 136302ACH, 173701ATB, 188102ATB, 203401ATB |

ACEi, angiotensin-converting enzyme inhibitor; ARB, angiotensin receptor blocker; BB, beta-blocker; CCB, calcium channel blocker; DM, diabetes mellitus; TCA, tricyclic antidepressant; SSRI, selective serotonin reuptake inhibitors; SNRI, serotonin and norepinephrine reuptake inhibitors; NRI, norepinephrine reuptake inhibitor; NaSSA, noradrenergic and specific serotonergic antidepressant; MAOi, monoamine oxidase inhibitor.

**Table S3.** TRIPOD Checklist.

| **Section/Topic** | **Item** | **Checklist Item** | **Page** |
| --- | --- | --- | --- |
| **Title and abstract** |  |  |  |
| Title | 1 | Identify the study as developing and/or validating a multivariable prediction model, the target population, and the outcome to be predicted. | 1 |
| Abstract | 2 | Provide a summary of objectives, study design, setting, participants, sample size, predictors, outcome, statistical analysis, results, and conclusions. | 3,4 |
| **Introduction** |  |  |  |
| Background and objectives | 3a | Explain the medical context (including whether diagnostic or prognostic) and rationale for developing or validating the multivariable prediction model, including references to existing models. | 5 |
|  | 3b | Specify the objectives, including whether the study describes the development or validation of the model or both. | 5 |
| **Methods** |  |  |  |
| Source of data | 4a | Describe the study design or source of data (e.g., randomized trial, cohort, or registry data), separately for the development and validation data sets, if applicable. | 6-8 |
|  | 4b | Specify the key study dates, including start of accrual; end of accrual; and, if applicable, end of follow-up. | 6-8 |
| Participants | 5a | Specify key elements of the study setting (e.g., primary care, secondary care, general population) including number and location of centres. | 6-8 |
|  | 5b | Describe eligibility criteria for participants. | 6 |
|  | 5c | Give details of treatments received, if relevant. | 6-8 |
| Outcome | 6a | Clearly define the outcome that is predicted by the prediction model, including how and when assessed. | 6-8 |
|  | 6b | Report any actions to blind assessment of the outcome to be predicted. | 6-8 |
| Predictors | 7a | Clearly define all predictors used in developing or validating the multivariable prediction model, including how and when they were measured. | 6-8 |
|  | 7b | Report any actions to blind assessment of predictors for the outcome and other predictors. | 6-8 |
| Sample size | 8 | Explain how the study size was arrived at. | 6 |
| Missing data | 9 | Describe how missing data were handled (e.g., complete-case analysis, single imputation, multiple imputation) with details of any imputation method. | 6 |
| Statistical analysis methods | 10a | Describe how predictors were handled in the analyses. | 7-8 |
|  | 10b | Specify type of model, all model-building procedures (including any predictor selection), and method for internal validation. | 7-8 |
|  | 10d | Specify all measures used to assess model performance and, if relevant, to compare multiple models. | 7-8 |
| Risk groups | 11 | Provide details on how risk groups were created, if done. | - |
| **Results** |  |  |  |
| Participants | 13a | Describe the flow of participants through the study, including the number of participants with and without the outcome and, if applicable, a summary of the follow-up time. A diagram may be helpful. | 6 |
|  | 13b | Describe the characteristics of the participants (basic demographics, clinical features, available predictors), including the number of participants with missing data for predictors and outcome. | 8 |
| Model development | 14a | Specify the number of participants and outcome events in each analysis. | 8 |
|  | 14b | If done, report the unadjusted association between each candidate predictor and outcome. | 8-9 |
| Model  specification | 15a | Present the full prediction model to allow predictions for individuals (i.e., all regression coefficients, and model intercept or baseline survival at a given time point). | 8-9 |
|  | 15b | Explain how to the use the prediction model. | 8-10 |
| Model performance | 16 | Report performance measures (with CIs) for the prediction model. | 8-10 |
| **Discussion** |  |  |  |
| Limitations | 18 | Discuss any limitations of the study (such as nonrepresentative sample, few events per predictor, missing data). | 12 |
| Interpretation | 19b | Give an overall interpretation of the results, considering objectives, limitations, and results from similar studies, and other relevant evidence. | 10-12 |
| Implications | 20 | Discuss the potential clinical use of the model and implications for future research. | 10-12 |
| **Other information** |  |  |  |
| Supplemental information | 21 | Provide information about the availability of Supplemental resources, such as study protocol, Web calculator, and data sets. | 1 |
| Funding | 22 | Give the source of funding and the role of the funders for the present study. | 1 |

We recommend using the TRIPOD Checklist in conjunction with the TRIPOD Explanation and Elaboration document.

**Table S4.** Baseline characteristics of the derivation and validation cohorts.

| Variables | Derivation cohort (n=176,273) | Validation cohort (n=75,546) | P value |
| --- | --- | --- | --- |
| Age, years | 65.7 ± 13.5 | 65.7 ± 13.6 | 0.9 |
| BMI, kg/m^2^ | 24.3 ± 3.7 | 24.3 ± 3.7 | 0.9 |
| Hemoglobin, g/dL | 12.5 ± 2.1 | 12.4 ± 2.1 | 0.3 |
| Waist circumference, cm | 84.7 ± 9.9 | 84.6 ± 9.9 | 0.4 |
| Leisure time physical activity, MET-min/week | 898.3 ± 1213.5 | 893.4 ± 1200.7 | 0.2 |
| CCI | 5.1 ± 3.3 | 5.1 ± 3.3 | 0.9 |
| Myocardial infarction | 10,443 (5.9) | 4511 (6) | 0.6 |
| Congestive heart failure | 45378 (25.7) | 19393 (25.7) | 0.7 |
| Peripheral vascular disease | 46506 (26.4) | 19879 (26.3) | 0.7 |
| Cerebrovascular disease | 40367 (22.9) | 17243 (22.8) | 0.7 |
| Dementia | 18804 (10.7) | 8009 (10.6) | 0.6 |
| Chronic pulmonary disease | 66112 (37.5) | 28673 (38) | 0.03 |
| Rheumatologic disease | 13478 (7.7) | 5727 (7.6) | 0.6 |
| Peptic ulcer disease | 57915 (32.9) | 24772 (32.8) | 0.8 |
| Mild liver disease | 73660 (41.8) | 31672 (41.9) | 0.5 |
| Diabetes without chronic complication | 93230 (52.9) | 39933 (52.9) | 0.9 |
| Diabetes with chronic complication | 63240 (35.9) | 27129 (35.9) | 0.9 |
| Hemiplegia, paraplegia | 4893 (2.8) | 2061 (2.7) | 0.5 |
| Any malignancy including leukemia and lymphoma | 32639 (18.5) | 13945 (18.5) | 0.7 |
| Moderate, severe liver disease | 4023 (2.3) | 1662 (2.2) | 0.2 |
| Metastatic solid tumor | 5475 (3.1) | 2353 (3.1) | 0.9 |
| AIDS | 249 (0.1) | 122 (0.2) | 0.2 |
| Age category |  |  | 0.9 |
| <65 years | 81030 (46) | 34731 (46) |  |
| >65 years | 95242 (54) | 40815 (54) |  |
| BMI category |  |  | 0.6 |
| <18.5 kg/m^2^ | 6464 (3.7) | 2812 (3.7) |  |
| 18.5–24.9 kg/m^2^ | 99063 (56.2) | 42299 (56) |  |
| 24.9–30 kg/m^2^ | 58901 (33.4) | 25401 (33.6) |  |
| >30 kg/m^2^ | 11845 (6.7) | 5034 (6.7) |  |
| CCI category |  |  | 0.8 |
| ≤7 | 137811 (78.2) | 59098 (78.2) |  |
| >7 | 38462 (21.8) | 16448 (21.8) |  |
| Hemoglobin category |  |  | 0.9 |
| ≤10 g/dL | 23003 (13.3) | 9844 (13) |  |
| >10 g/dL | 153270 (87) | 65702 (87) |  |
| Sex |  |  | 0.3 |
| Male | 104885 (59.5) | 44769 (59.3) |  |
| Female | 71388 (40.5) | 30777 (40.7) |  |
| Household income |  |  | 0.08 |
| Quantile 1 | 42740 (24.3) | 18402 (24.4) |  |
| Quantile 2 | 30476 (17.3) | 12745 (16.9) |  |
| Quantile 3 | 39694 (22.5) | 17146 (22.7) |  |
| Quantile 4 | 63363 (36) | 27253 (36.1) |  |
| Residential area |  |  | 0.7 |
| Urban | 101227 (57.4) | 43313 (57.3) |  |
| Rural | 75046 (42.6) | 32233 (42.7) |  |
| History of hospitalization | 125301 (71.1) | 53788 (71.2) | 0.6 |
| History of ED admission | 41854 (23.7) | 17839 (23.6) | 0.5 |
| Psychiatric clinic | 3275 (1.9) | 1462 (1.9) | 0.2 |
| Depression | 75503 (42.8) | 32567 (43.1) | 0.2 |
| Insomnia | 72639 (55.6) | 31056 (55.5) | 0.3 |
| Suicide attempt | 2388 (1.4) | 1052 (1.4) | 0.5 |
| Atrial fibrillation | 12389 (7) | 5398 (7.2) | 0.3 |
| Smoking | 32828 (18.6) | 13918 (18.4) | 0.2 |
| Drinking | 49161 (27.9) | 20819 (27.6) | 0.09 |
| Diabetes mellitus | 139819 (79.3) | 59693 (79) | 0.09 |
| Hypertension | 161129 (91.4) | 69100 (91.5) | 0.6 |
| Anxiety disorder | 90097 (51.1) | 38488 (51) | 0.4 |
| Psychosis | 7162 (4.1) | 3145 (4.2) | 0.2 |
| Substance misuse | 7555 (4.3) | 3176 (4.2) | 0.4 |
| PTSD | 395 (0.2) | 162 (0.2) | 0.6 |
| Bipolar disorder | 11237 (6.4) | 4882 (6.5) | 0.4 |
| Schizophrenia, schizophrenic affective disorder | 4475 (2.5) | 1995 (2.6) | 0.1 |
| Amputation | 2432 (1.4) | 967 (1.3) | 0.05 |
| Alcohol abuse | 703 (0.4) | 274 (0.4) | 0.2 |
| Drug abuse | 29218 (16.6) | 12476 (16.5) | 0.7 |
| CRPS | 3589 (2) | 1494 (2) | 0.3 |
| Myocardial infarction | 24240 (13.8) | 10275 (13.6) | 0.3 |
| Stroke | 50156 (28.5) | 21485 (28.4) | 0.9 |
| MACE | 63204 (35.9) | 27039 (35.8) | 0.8 |
| Liver cirrhosis | 17173 (9.7) | 7312 (9.7) | 0.6 |

AIDS, acquired immune deficiency syndrome; BMI, body mass index; CCI, Charlson comorbidity index; CRPS, complex regional pain syndrome; ED, emergency department; ESKD, end-stage kidney disease; ICU, intensive care unit; MACE, major adverse cardiovascular event; METs, metabolic equivalent of tasks; PTSD, post-traumatic stress disorder.

**Table S5.** Univariate hazard ratio and C-index for suicide risk by variables.

| Variables | Univariate HR | | C-index |
| --- | --- | --- | --- |
|  | HR (95% CI) | P-value |  |
| Age (years) | 1.01 (1.01–1.02) | <.001 | 0.5391 |
| METs category |  |  | 0.5289 |
| No physical activity | 1 (ref.) |  |  |
| <500 MET-min/week | 0.75 (0.61–0.91) | 0.003 |  |
| 500–999 MET-min/week | 0.82 (0.68–1) | 0.05 |  |
| ≥1000 MET-min/week | 0.79 (0.67–0.94) | 0.008 |  |
| BMI category |  |  | 0.5557 |
| <18.5 kg/m^2^ | 1 (ref.) |  |  |
| 18.5–24.9 kg/m^2^ | 1.03 (0.72–1.45) | 0.9 |  |
| 24.9–30 kg/m^2^ | 0.86 (0.60–1.23) | 0.4 |  |
| >30 kg/m^2^ | 0.63 (0.44–0.89) | 0.009 |  |
| CCI category |  |  | 0.5144 |
| ≤7 | 1 (ref.) |  |  |
| >7 | 0.86 (0.69–1.07) | 0.2 |  |
| Hb category |  |  | 0.5321 |
| ≤10 g/dL | 1 (ref.) |  |  |
| >10 g/dL | 1.93 (1.47–2.53) | <.001 |  |
| Sex |  |  | 0.5683 |
| Male | 1 (ref.) |  |  |
| Female | 0.52 (0.45–0.61) | <.001 |  |
| Household income |  |  | 0.5191 |
| Quantile 1 | 1 (ref.) |  |  |
| Quantile 2 | 1.11 (0.9–1.37) | 0.3 |  |
| Quantile 3 | 1.17 (0.96–1.43) | 0.1 |  |
| Quantile 4 | 1.07 (0.89–1.28) | 0.5 |  |
| Residential area |  |  | 0.5925 |
| Urban | 1 (ref.) |  |  |
| Rural | 2.10 (2.46–1.81) | <.001 |  |
| History of hospitalization | 1.26 (1.08–1.45) | 0.03 | 0.5165 |
| History of ED admission | 1.19 (1–1.41) | 0.05 | 0.5115 |
| History of ICU admission | 1.14 (0.96–1.36) | 0.1 | 0.5009 |
| Psychiatric clinic | 1.3 (0.83–2.02) | 0.3 | 0.5011 |
| Depression | 1.84 (1.6–2.1) | <.001 | 0.5735 |
| Insomnia | 1.74 (1.51–2.01) | <.001 | 0.5582 |
| Suicide attempt | 1.96 (1.13–3.39) | 0.02 | 0.5048 |
| Atrial fibrillation | 0.96 (0.69–1.33) | 0.8 | 0.5036 |
| Smoking | 1.9 (1.63–2.21) | <.001 | 0.5541 |
| Drinking | 1.55 (1.35–1.79) | <.001 | 0.555 |
| No diabetes mellitus | 0.8 (0.7–0.93) | 0.003 | 0.5437 |
| No hypertension | 0.33 (0.29–0.39) | <.001 | 0.5995 |
| Anxiety disorder | 1.5 (1.31–1.72) | <.001 | 0.5446 |
| Psychosis | 2.49 (1.91–3.26) | <.001 | 0.5214 |
| Substance misuse | 3.62 (2.91–4.51) | <.001 | 0.5366 |
| PTSD | 3.67 (1.53–8.85) | 0.004 | 0.5023 |
| Bipolar disorder | 2.26 (1.74–2.94) | <.001 | 0.5199 |
| Schizophrenia, schizophrenic affective disorder | 2.71 (1.99–3.7) | <.001 | 0.5167 |
| Amputation | 1.09 (0.56–2.09) | 0.8 | 0.4998 |
| Alcohol abuse | 5.77 (3.33–9.97) | <.001 | 0.506 |
| Drug abuse | 1.98 (1.69–2.32) | <.001 | 0.545 |
| CRPS | 0.84 (0.45–1.57) | 0.6 | 0.5009 |
| Myocardial infarction | 0.82 (0.64–1.07) | 0.1 | 0.5103 |
| Stroke | 1.13 (0.97–1.33) | 0.1 | 0.5052 |
| MACE | 1.1 (0.95–1.27) | 0.2 | 0.5022 |
| Liver cirrhosis | 1.08 (0.84–1.38) | 0.6 | 0.5019 |

AIDS, acquired immune deficiency syndrome; BMI, body mass index; CCI, Charlson comorbidity index; CRPS, Complex regional pain syndrome; ED, emergency department; ESKD, end-stage kidney disease; ICU, intensive care unit; MACE, major adverse cardiovascular event; METs, metabolic equivalent of tasks; PTSD, post-traumatic stress disorder.

**Table S6. Predictive performance of the suicide risk score for patients with ESKD.**

|  | Optimism corrected c-index | 95% CI |
| --- | --- | --- |
| Validation cohort |  |  |
| 1 year | **0.716** | **0.695-0.737** |
| 3 year | **0.714** | **0.694-0.734** |
| 5 year | **0.711** | **0.686–0.736** |
| 10 year | **0.709** | **0.697–0.721** |

**CI, confidence intervals.**

**Table S7.** The other suicide risk score using minimal variables (AUC: 0.681).

| Clinical risk prediction tool | Scores  46 full |
| --- | --- |
| Age |  |
| <65 years | 0 |
| ≥65 years | 1 |
| BMI |  |
| <18.5 kg/m^2^ | 7 |
| 18.5-24.9 kg/m^2^ | 6 |
| 25–29.9 kg/m^2^ | 5 |
| >30 kg/m^2^ | 0 |
| CCI |  |
| ≤7 | 0 |
| >7 | 3 |
| Hb category |  |
| ≤10 g/dL | 0 |
| >10 g/dL | 3 |
| Sex |  |
| Male | 5 |
| Female | 0 |
| Residential area |  |
| Urban | 0 |
| Rural | 3 |
| Depression | 3 |
| Insomnia | 3 |
| Smoking | 2 |
| Suicide attempt | 3 |
| Anxiety disorder | 1 |
| Psychosis | 3 |
| Alcohol abuse | 8 |
| Physical activity (yes) | 1 |

**Table S8.** The other suicide risk scores using other variables (AUC: 0.694).

| Clinical risk prediction tool | Scores  25 full |
| --- | --- |
| Age |  |
| <65 years | 0 |
| ≥65 years | 1 |
| BMI |  |
| <18.5 kg/m^2^ | 7 |
| 18.5-24.9 kg/m^2^ | 7 |
| 25–29.9 kg/m^2^ | 5 |
| >30 kg/m^2^ | 0 |
| Sex |  |
| Male | 5 |
| Female | 0 |
| Residential area |  |
| Urban | 0 |
| Rural | 3 |
| Depression | 1 |
| Insomnia | 3 |
| Psychosis | 2 |
| Alcohol abuse | 3 |

Total score range: 0-25.


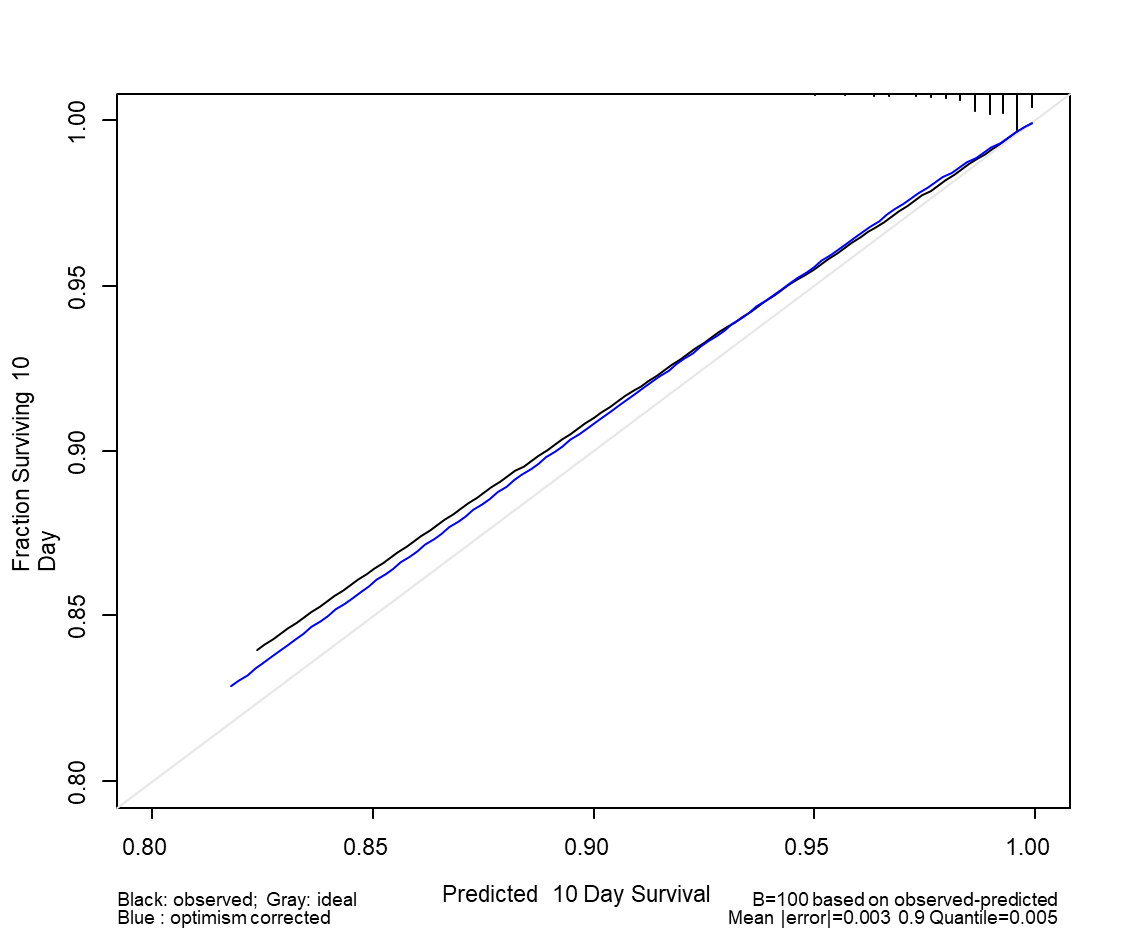
**Figure S1.** Calibration plot for suicide. Calibration of the clinical risk prediction score in the validation cohort is presented. The x-axis represents the predicted 10-year event rate without suicide events. The Y-axis represents the actual 10-year suicide rate. The black line represents perfect calibration, and the blue line indicates the observed optimism-corrected calibration. Optimism correction was performed using bootstrapping 100 resamples.
